# Restoration and risk reduction of lead mining waste by phosphate-enriched biosolid amendments

**DOI:** 10.1038/s41598-021-88576-y

**Published:** 2021-04-26

**Authors:** Na Li, Xi Tang, John Yang, Zhanxiang Sun

**Affiliations:** 1 Liaoning Dry Land Agricultural and Forestry Research Institute, Chaoyang, 122000 China; 2 Department of Agriculture and Environmental Science and Cooperative Research, Lincoln University of Missouri, Jefferson City, MO 65101 USA; 3 Liaoning Academy of Agriculture Science, Shenyang, 110866 China

**Keywords:** Ecology, Environmental sciences, Natural hazards

## Abstract

Lead (Pb) contamination in environment has been identified as a threat to human health and ecosystems. In an effort to reduce the health and ecological risks associated with Pb mining wastes, a field study was conducted to stabilize Pb using phosphate (P)-enriched biosolid amendments in the contaminated mining wastes (average of 1004 mg Pb kg^−1^) located within the Jasper County Superfund Site, southwest Missouri. Experiments consisted of six biosolid amendment treatments, including Mizzou Doo compost (MD); Spent mushroom compost (SMC); Turkey litter compost (TLC); Composted chicken litter (CCL); Composted sewage sludge (CSS); and Triple superphosphate (TSP). Kentucky tall fescue seeds were planted following the treatments, and soil and plant samples were collected and analyzed 8–10 years post treatment. Results indicated that, in all cases, the biosolid treatments resulted in significant reductions in bioaccessible Pb (96.5 to 97.5%), leachable Pb (95.0 to 97.1%) and plant tissue Pb (45.5 to 90.1%) in the treated wastes, as compared with the control. The treatments had no significantly toxicological effect to soil microbial community. Analysis of the Pb fractionation revealed that the Pb risk reduction was accomplished by transforming labile Pb fractions to relatively stable species through the chemical stabilization reactions as induced by the treatments. The solid-phase microprobe analysis confirmed the formation of pyromorphite or pyromorphite-like minerals after the treatment. Among the six biosolid amendments examined, SMC and MD treatments were shown most effective in the context of Pb stabilization and risk reduction. This field study demonstrated that the treatment effectiveness of Pb stabilization and risk reduction in mining wastes by P-enriched biosolid amendments was long-term and environmental-sound, which could be potentially applied as a cost-effective remedial technology to restore contaminated mining site and safeguard human health and ecosystems from Pb contamination.

## Introduction

Heavy metal contamination in natural environments such as lead (Pb) resulting from mining operations or various human activities has been identified as a serious threat to human health and ecosystem because of its human toxicity and persistence in the environment^1^. In Jasper County, southwestern Missouri of the United States of America, a survey indicated that 14% of children under the age of 7 years living in this mining community were found to have an elevated blood Pb level (higher than 100 µg L^−1^). This was identified to be directly associated with Pb contamination in soils and mining wastes resulting from mining or smelting activities^2^. The Pb contamination in the environment has also shown adverse impacts on wildlife^3^, surface and groundwater quality, and vegetation^4^.

Lead toxicological risk to human health was proportional to Pb bioavailability, which is the ability of Pb to be dissolved in the gastrointestinal (GI) tract and absorbed into the blood system through oral ingestion, while the Pb ecological risk was related to the Pb dissolution in water and mobility in ecosystems^5,6^. Both risks are strongly associated with the Pb dissolution in the GI tract and water and highly dependent on the chemical or mineralogical forms of Pb and their solubility. It was believed that the transformation of Pb from labile species to insoluble forms would substantially reduce the Pb health and ecological risks.

In situ soil treatment using phosphate or organic materials has been evaluated as a cost-effective and environmental-sound remedial technology for immobilizing or stabilizing heavy metals in contaminated soils and reducing the human health and ecological risks. Pyromorphites [Pb_5_(PO_4_)_3_ (OH, Cl, F…)] or pyromorphite-like minerals were known biologically stable and chemically insoluble under the surface soil conditions and relatively non-bioavailable to human^7^. Previous studies of soil Pb remediation have been primarily focused on inducing the transformation of labile soil Pb to pyromorphites by various phosphate-based amendments, including phosphate rocks^8^, synthetic hydroxapatite^9,10^, monopotassium phosphate^11^, and phosphoric acid^12–14^ and proven effective for soil Pb immobilization and risk reduction. The formation of pyromorphites in Pb-contaminated soil as induced by phosphate amendments has been confirmed^6,8,12^. Applications of biosolids such as municipal sewage sludge, composts, manures, or peats have also shown to effectively stabilize Pb and reduce the Pb bioavailability in soil through the mechanisms of immobilization, adsorption or complexation reactions^15–18^. As said, in situ Pb immobilization was an approach that converts soil Pb to relatively insoluble Pb species induced by amendments, without Pb removal from soil ecosystem. The chemically and biologically stable Pb species formed after treatment would be the key for assessing the efficacy of this remedial technology. Thus, the long-term assessment of the immobilized Pb stability and treatment effectiveness under field conditions, specifically plant growing soil conditions, would be critically needed for ensuring the success of this cost-effective technology and its large-scale application.

In an effort to restore or remediate the Pb-contaminated mining waste sites in the Jasper County Superfund Site of the historical tri-state lead mining district, southwestern Missouri. The mining wastes were treated with six P-enriched biosolid amendments. The overall goal of this presented demonstration study was to assess the treatment effectiveness and evaluate their long-term efficacy for stabilizing Pb and reducing the human health and ecological risks of the mining wastes post 8–10 year treatments. Specific objectives were to: (1) assess the health risk reduction by the biosolid treatments through in vitro bioavailability test; (2) evaluate the mobility or stability of the stabilized Pb by leachability assessment; (3) determine the treatment impacts on plant Pb uptake with plant tissue analysis; (4) investigate the toxicological effect of the amendments to soil microbes using bacteria-based assay; and (5) examine the Pb species responsible for the risk reduction by sequential chemical fractionation and solid-phase speciation analyses.

## Materials and methods

### Site description

**Figure1:**
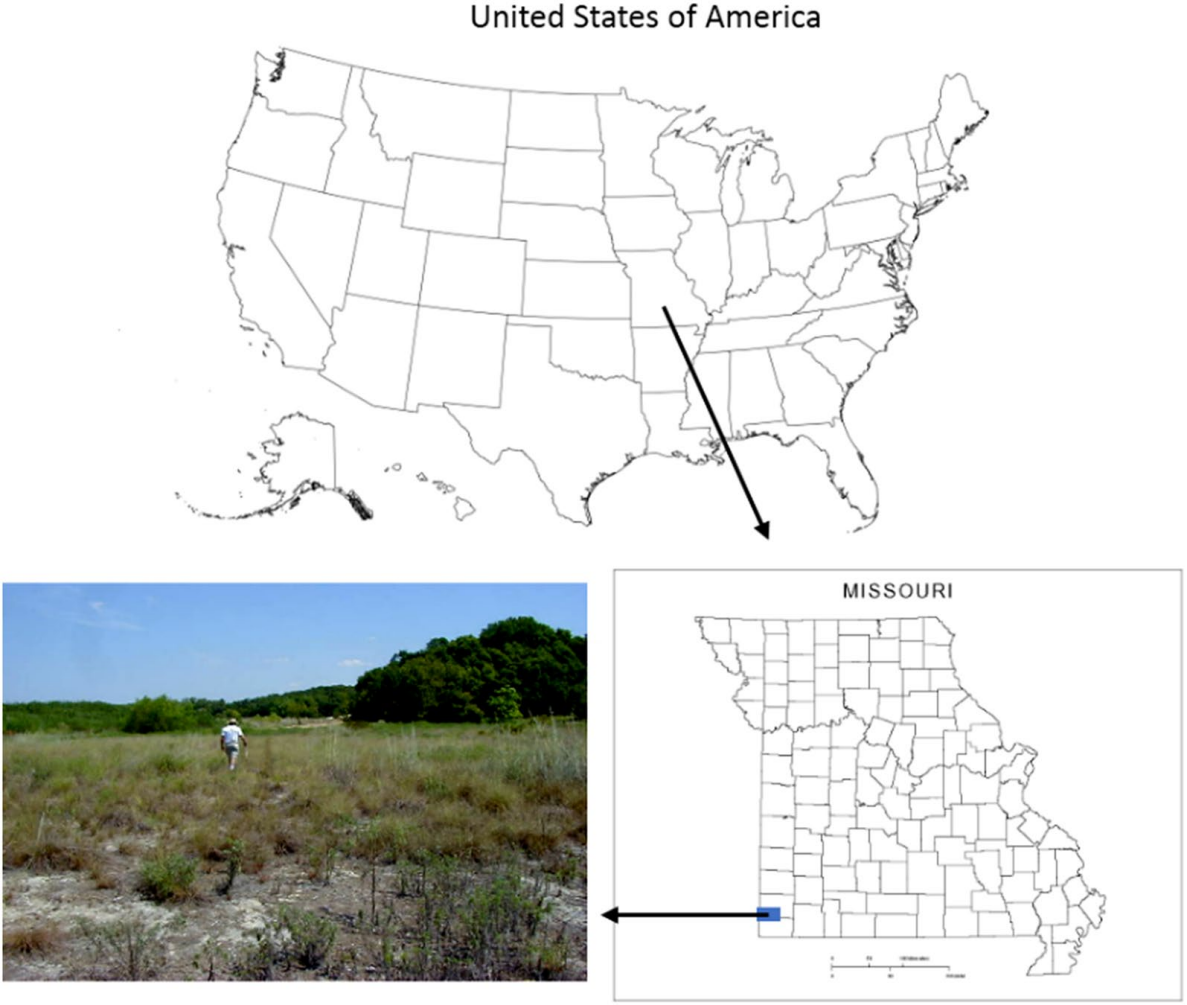
Location and photo of the study site (USA map https://www.pinterest.com/pin/305330049740108110/ ; MO map source: http://www.yellowmaps.com/map/missouri-blank-map-115.htm).

An abandoned mining site in the rural area located 1.6 km north of Webb City within the Jasper County Superfund Site of southwestern Missouri was selected for this study as shown in Fig. 1. The site had an area of 22.3 ha, with 18.2 ha on floodplain and 4.1 ha on uplands (2–3% slope) of Center Creek, and was completely or mostly barren with a few islands of vegetation occurring along small waterways and depressions where surface water runoff accumulated. The area was under a humid subtropical climate, with annual average temperature of 14.7 °C and annual average precipitation of 1182 mm.

The mining wastes on the site primarily consisted of a mixture of chats and tailings ranging from fine sand to gravel (0.6 cm diameter) in size and contained an average of 1004 mg Pb kg^−1^, with spatial variations ranging from 230 to 3800 mg Pb kg^−1^ across the site, as determined by the USEPA standard x-ray fluorescence method using Innov-X portable XRF analyzer. Lead carbonate (PbCO_3_), a relatively soluble and highly bioavailable Pb species, was identified as a primary solid Pb mineral in the wastes by x-ray diffraction (Rigaku desktop Min-Flex XRD). Tailings, fine mining waste, were found containing higher Pb contents than chats. In addition, the waste also contained average of 3492 mg Zn kg^−1^ and 68 mg Cd kg^−1^. Detailed characterization of the mining wastes could be found in the report by Mosby et al.^19^.

### Experimental procedures

Six soil amendments used for treatments included (1) Mizzou Doo compost (MD), a locally manufactured proprietary blend of animal waste, post consumer paper, sawdust and other patented ingredients (0.08% P); (2) Spent mushroom compost (SMC, 0.36% P); (3) Composted sewage sludge (CSS), municipal sewage sludge composted with sawdust (1.93% P); (4) Composted chicken litter (CCL), chicken litter composted with sawdust and peanut skins (1.73% P); (5) Turkey litter compost (TLC, 1.5% P); and (6) Triple super phosphate (TSP). Physiochemical characterization of the amendments can be found in the report by Mosby et al.^19^. The treatment with selected biosolid materials attempted to improve the physiochemical properties of the mining wastes and facilitate the vegetation establishment on the site, while the enriched P content intended to enhance Pb stabilization or immobilization on the mining wastes.

The field experiment consisted of seven 2- to 6-ha plots randomly arranged across the site. The five biosolid amendments were applied, one per each plot, at a rate of 123 to 247 tons (dry weight) per ha, based on the Pb concentration measured in the wastes, and TSP was applied in the plot containing high Pb content at a rate of 3.2 tons per ha. Untreated plot was included and served as control. The treatment plots were established in early 1999 as a demonstration site without replications per treatment, because of the large-size of the plots. The amendments applied in each plot were incorporated into the top 15-cm wastes by a tiller, and the grass seeds of Kentucky tall fescue planted one week post treatment for vegetation establishment on the site. The plots had not been irrigated, and no grass biomass harvested since the plot establishment.

Topsoils (0–15 cm) and above-ground plant tissue were collected from three randomly-selected spots within each treated plot, representing three replicated samples of the treatment. Both soil and plant samples were collected every 3-month interval for 2 years post 8 year treatment. The collected soil samples were air-dried and ground to pass through a 0.25 mmsieve, while the plant samples were oven-dried at 70 °C for 24 h and ground to pass through a 0.1 mm sieve. Both samples were stored at room temperature until analysis.

### Analytical procedures

Bioaccessible Pb is the measurement that assesses the potential exposure risk of soil Pb to human health based on the modified physiology-based extraction test (PBET) that simulates Pb dissolution in the GI tract. Analyses were performed following the procedures as described by Yang et al.^12^. Leachable Pb, an assessment of potential risk of soil Pb to water quality or chemical stability of soil Pb, was determined using the procedures described by Singh et al.^20^. Plant tissue Pb, an indication of phytoavailability or the risk of soil Pb to plant community, was measured following the procedures of the USEPA Methods 3050. Microtox test, a bacteria-based assay that estimates the soil toxicity to microbial community, was analyzed with the procedures by Yang et al.^21^.

Sequential chemical extraction fractionation of soil Pb, including water-soluble, exchangeable, carbonate, Fe/Mn oxide, organic, and residue fractions^22^, was performed to investigate the Pb transformation as induced by treatments and identify the chemical species responsible for the Pb immobilization or risk reduction. The solubility of the six Pb fractions extracted was from the most soluble to relatively insoluble. In an effort to confirm the formation of solid pyromorphites, solid-phase Pb speciation was examined with the microprobe analysis using scanning electron microscopy combined with energy dispersive spectroscopy (Hitachi Amray-1600T SEM–EDS) as described by Yang et al.^12^.

Inductively coupled plasma–optical emission spectroscopy (Varian Vista-Pro ICP-OES) was used to determine Pb concentration in the soil or plant samples. All analyses were conducted in triplicate per sample, with recalibration every 15-sample run using a standard reference material, NIST-SRM 1640 Trace Elements in Natural Water. A synthetic chloropyromorphite standard was included in the analyses for the solid-phase speciation.

### Statistical analysis

Data analysis was performed by standard analysis of variance (ANOVA) for mean comparisons among treatments, sampling time, and treatment x time interactions using the procedure in Statistix 8.1 software (Analytical Software Co.). Critical values (CV) and least significant differences (LSD) were calculated to separate means among treatments at the 0.05 significance level (*P* < *0.05*).

## Results and discussion

### Health risk reduction

**Figure 2:**
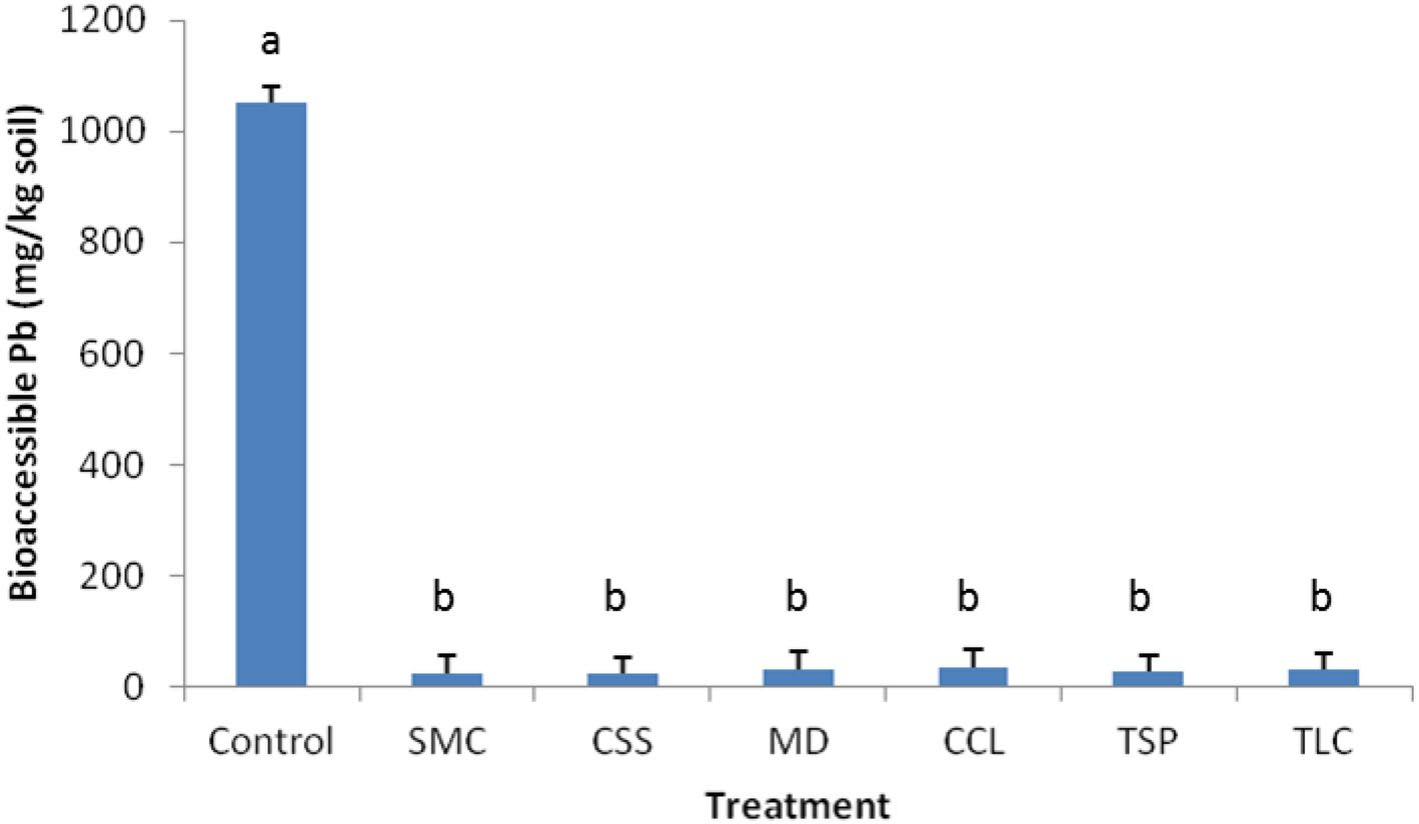
Average of bioaccessible Pb in the amendment-treated and untreated (control) mining wastes during the 8–10 year sampling period post treatment. Vertical bars indicate the standard deviation of measurements, and different letters denote significant difference (*p* < 0.05) among treatments.

As mentioned above, bioaccessible Pb represented the potential toxicity or exposure risk of soil Pb to human health based on the modified physiology-based extraction test (PBET). Measurements presented in Fig. 2 showed that, in all cases, Pb bioaccessibility in the mining wastes was significantly reduced by the amendment treatments compared to untreated control (*p* < 0.05). This finding was consistent with previous studies reported^18,23^, where similar biosolid treatments resulted in a significant reduction of Pb bioavailability in Pb-contaminated soils. The averaged reductions by the amendments were in the order: CSS (97.8%) > SMC (97.6%) > TSP (97.4%) > TLC (97.0%) > MD (96.8%) > CCL (96.5%), which was found not significantly different among the treatments and during the sampling period. The concentrations of bioacessible Pb in control plot (averaged 1050 mg Pb kg^−1^) was found slightly higher than the average of total Pb content (1004 mg Pb kg^−1^) on the site determined by the standard XRF method. This could be due to sampling variation and spatial Pb variability on the site. The wastes were heterogeneous across the site (230–3800 mg Pb kg^−1^) and consisted of a mixture of chat gravels and tailing sands, with about 10% sample volume passing through a 0.25 mm sieve for analysis. The fine particles usually contained higher Pb content than chat and large particles. Thus the samples that were collected and analyzed could contain higher Pb than the average concentration in the wastes. Even under the assumption that all > 0.25 mm sample particles contained no or little Pb, the reductions of bioaccessible Pb by the treatments were still estimated at a range from 47.5 to 92.1%. The treatment effectiveness was found similar to the results on the Pb milling waste study reported by Tang and Yang^14^.

The significant reduction of Pb bioaccessibility or potential health risks in the mining wastes could have resulted from the Pb transformation from labile or bioaccessible forms to less soluble or relatively stable species as induced by the amendment treatments through immobilization reactions. The reaction mechanisms could include formation of pyromorphites or pyromorphite-like minerals; chelation by organic function groups; and surface adsorption or complexation by organic materials^12,24^, which made the stabilized Pb less dissolvable in the extracting solution. In addition, the measurements post 8–10 years treatments indicated that the transformed Pb species were relatively stable under the surface soil conditions and the treatment effectiveness was maintained in long-term, with less likely being dissolved in the GI tract and absorbed into the human bloodstream.

### Ecological risk reduction

The ecological risk assessments used in this study included the measurement of the Pb uptake by plants, Pb potential leachability to groundwater, and toxicity to soil microbial communities.

**Figure 3:**
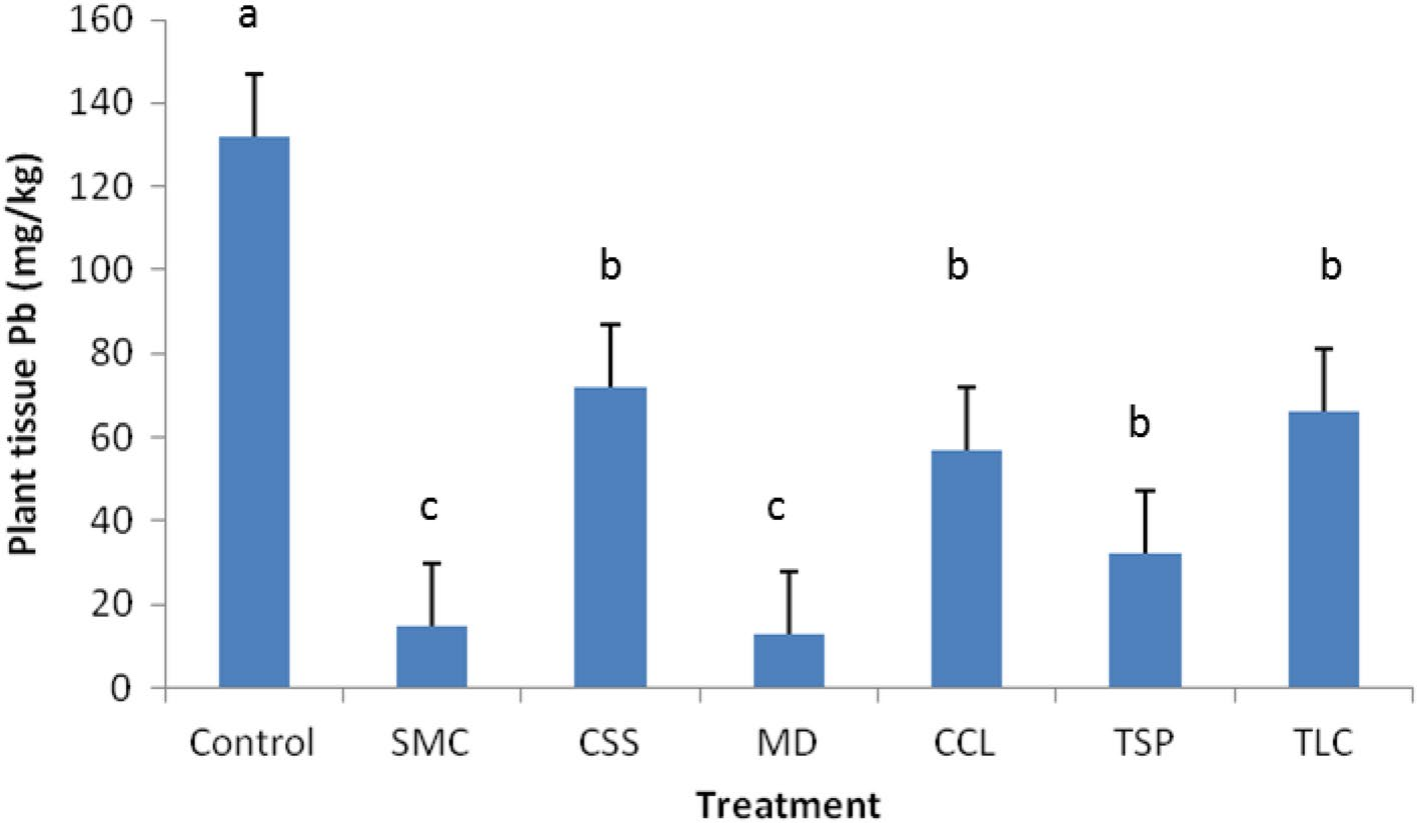
Average of plant tissue Pb in the amendment-treated and untreated (control) mining wastes during the 8–10 year sampling period post treatment. Vertical bars indicate the standard deviation of measurements, and different letters denote significant difference (*p* < 0.05) among treatments.

After one year post treatments, tall fescue grasses started growing in the treated plots, with about 70–80% plant coverage estimated, but none or very few grasses were grown in control plot (no data collected). The grass establishment on the treated plots could be due to improved physiochemical properties of the mining wastes by the applied biosolid amendments, such as water holding capacity and nutrient status, and reduced Pb toxicity to plants through the immobilization processes. Analyses of the above-ground plant tissues indicated that all amendments significantly lowered Pb uptake by the grasses (Fig. 3, *p* < 0.05). Tissue Pb concentrations were in the order: CSS (72 mg Pb kg^−1^) > TLC > CCL > TSP > SMC > MD (13 mg Pb kg^−1^). In compared with the control plot (132 mg Pb kg^−1^), the reductions by the treatments ranged from 45 to 90%, similar to the measurements of bioaccessible Pb. Reduced plant Pb uptake could be achieved by phytoavailability reduction of soil Pb species induced by the biosolid treatments^25,26^. However, no significant correlation was found between Pb bioaccessibility and plant uptake. This could be attributed to difference in solubility of the Pb species formed. The Pb species formed through different stabilizing mechanisms by various organic biosolids may dissolve differently under the GI and rhizosphere conditions.

**Figure 4:**
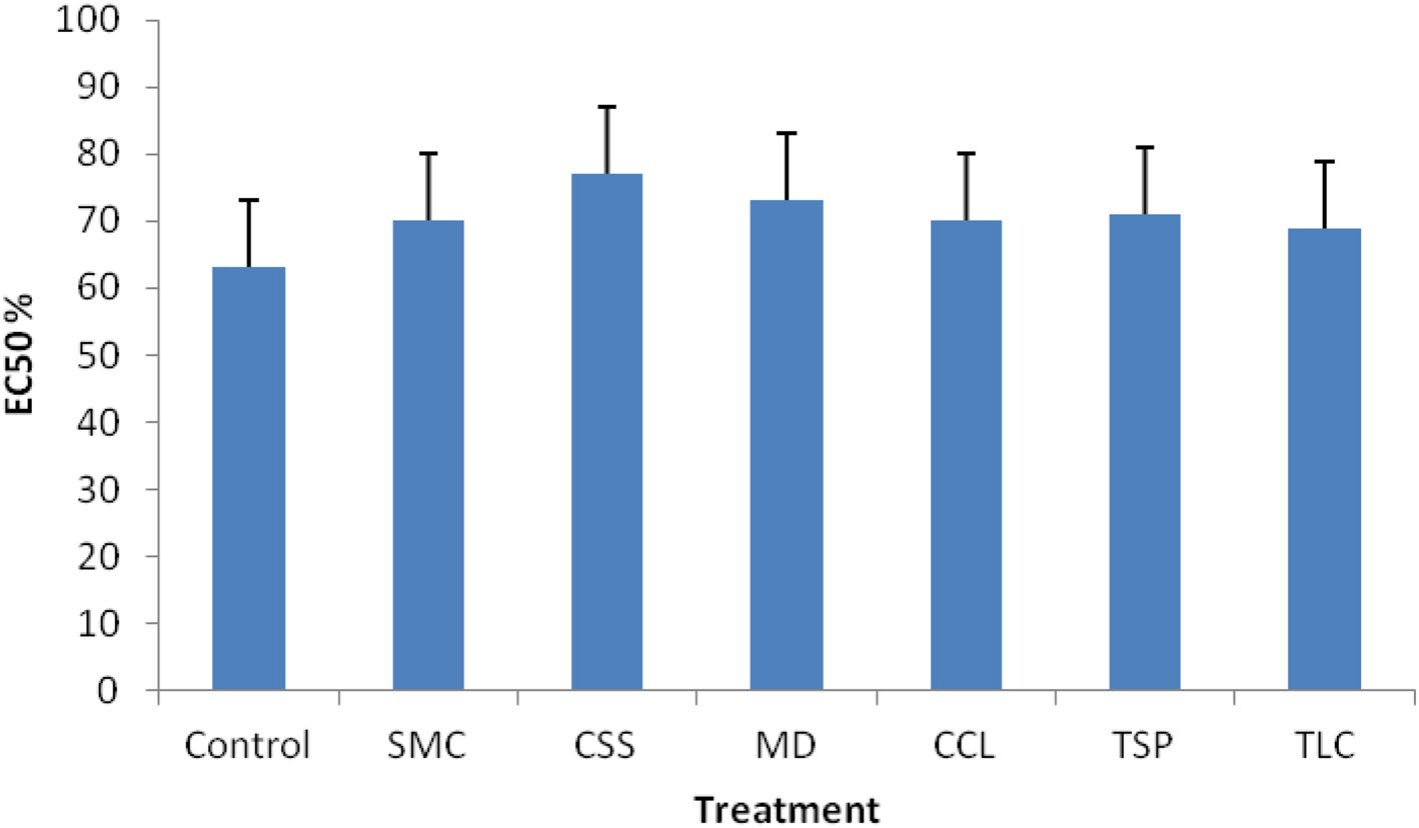
Average of soil microbial toxicity in the amendment-treated and untreated (control) mining wastes during the 8–10 year sampling period post treatment. Vertical bars indicate the standard deviation of measurements.

The microbial toxicity assay (Microtox test) was used to assess the potential toxicological risk of the soils to microbial communities. The assay consisted in the measurement of the effective concentration of soil solution that caused 50% death of bioluminescent *V. fischeri* bacteria (EC50%), based on bioluminescence reduction compared to “non-toxic” conditions^27^. Higher EC50% value would represent a lower toxicity or lower risk to soil microbial community. The assay measurements presented in Fig. 4 indicated that all amendment treatments resulted in slightly higher EC50% values (69–77%) or lower toxicological effects to soil microbial communities than control (63%), but the differences were not statistically significant (*p* > 0.05). The relatively higher EC50% values in treated plots suggested that the amendments would lower the toxicity to soil microbial community through the stabilization reactions and the creation of soil conditions that facilitated microbial activity and growth, due to the addition of organic matter and improved soil properties and nutrient status in treated mining wastes. Data illustrated that there was no adverse impact of the amendments on soil microbial community, and soil microbial activities and functions were maintained in the long-term after treatments.

**Figure 5:**
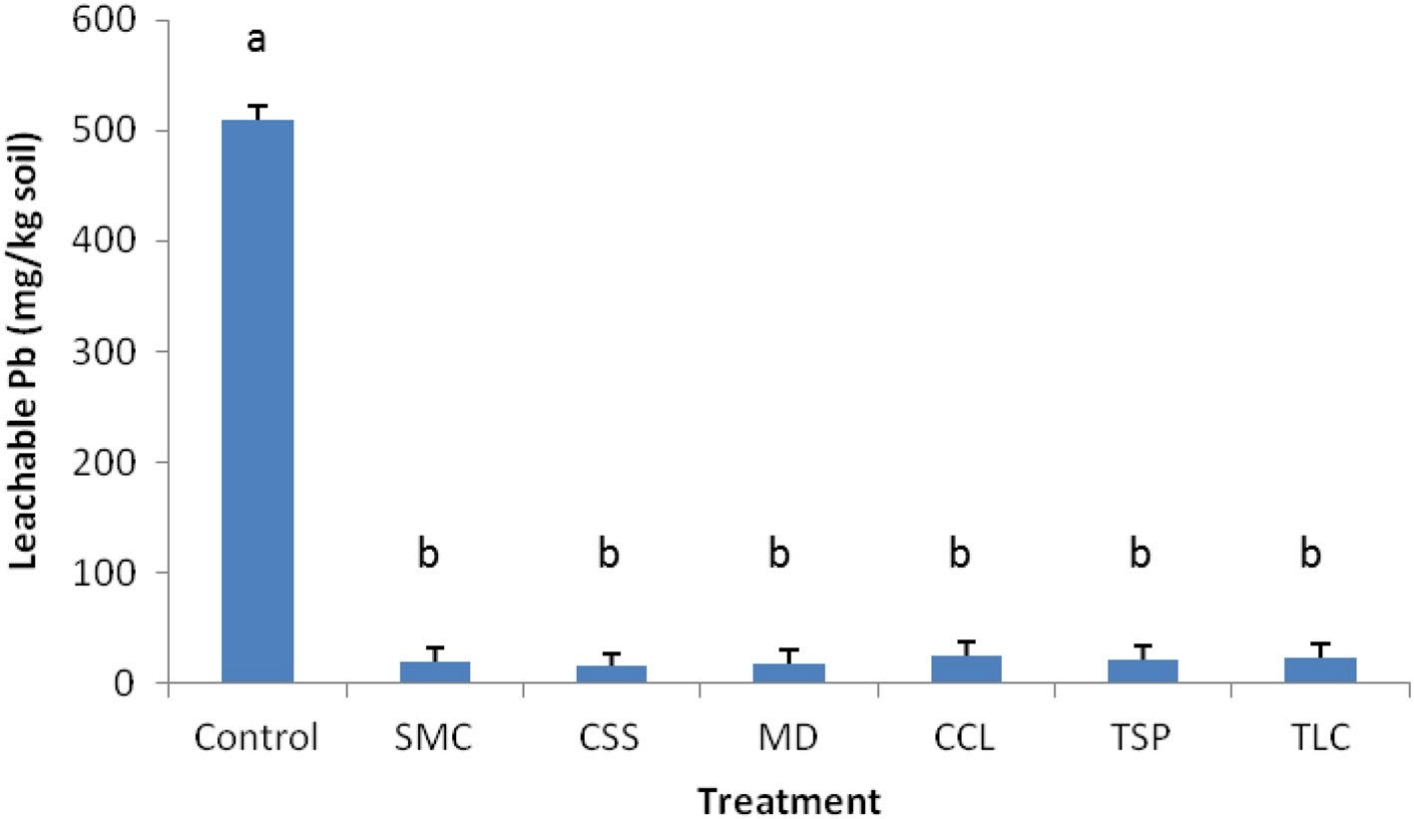
Average of leachable Pb in the amendment-treated and untreated (control) mining wastes during the 8–10 year sampling period post treatment. Vertical bars indicate the standard deviation of measurements, and different letters denote significant difference (*p* < 0.05) among treatments.

Measurement of Pb leachability represented the maximum amounts of soil Pb potentially leachable to surface and groundwater or the risk to aquatic ecosystem^20^. Data presented in Fig. 5 revealed that Pb leachability in all treated plots was significantly reduced by over 90% (*p* < 0.05) compared to averaged 500 mg Pb kg^−1^ in the control plots, which had a similar trend of Pb bioaccessibility measured (Fig. 2). The reductions by treatments varied a little and did not significantly differ across the sampling period (*p* > 0.05). The average of leachable Pb concentrations was in the order: CCL > TLC > TSP > SMC > MD > CSS. Reduced leachable Pb by the biosolids implied that much less Pb from the treated wastes would be leachable to surface and groundwater, consequently lowering the risk to aquatic ecosystem and protecting water quality near the mining site. This finding was consistent with data reported on the mill wastes treated by phosphoric acid^13^. As previously mentioned, the substantial reduction of Pb leachability by the biosolids could be caused by the formation of less soluble Pb species induced by the treatments through various stabilization mechanisms^28,29^. The Pb leachability was found much lower than the Pb bioaccessibility measured. This discrepancy could be relevant to acidity of the extracting solutions, in which pH 4 solution was used for Pb leachability measurement and pH 2 solution for Pb bioaccessibility. It was known that soil extractable Pb was dependent on chemical forms or solubility of Pb species, and Pb dissolution from soil was proportional to acidity of extraction solution^30,31^. As expected, more Pb would be extracted from soil by a pH 2 solution than a pH 4 solution.

### Alteration of Pb fraction

**Figure 6:**
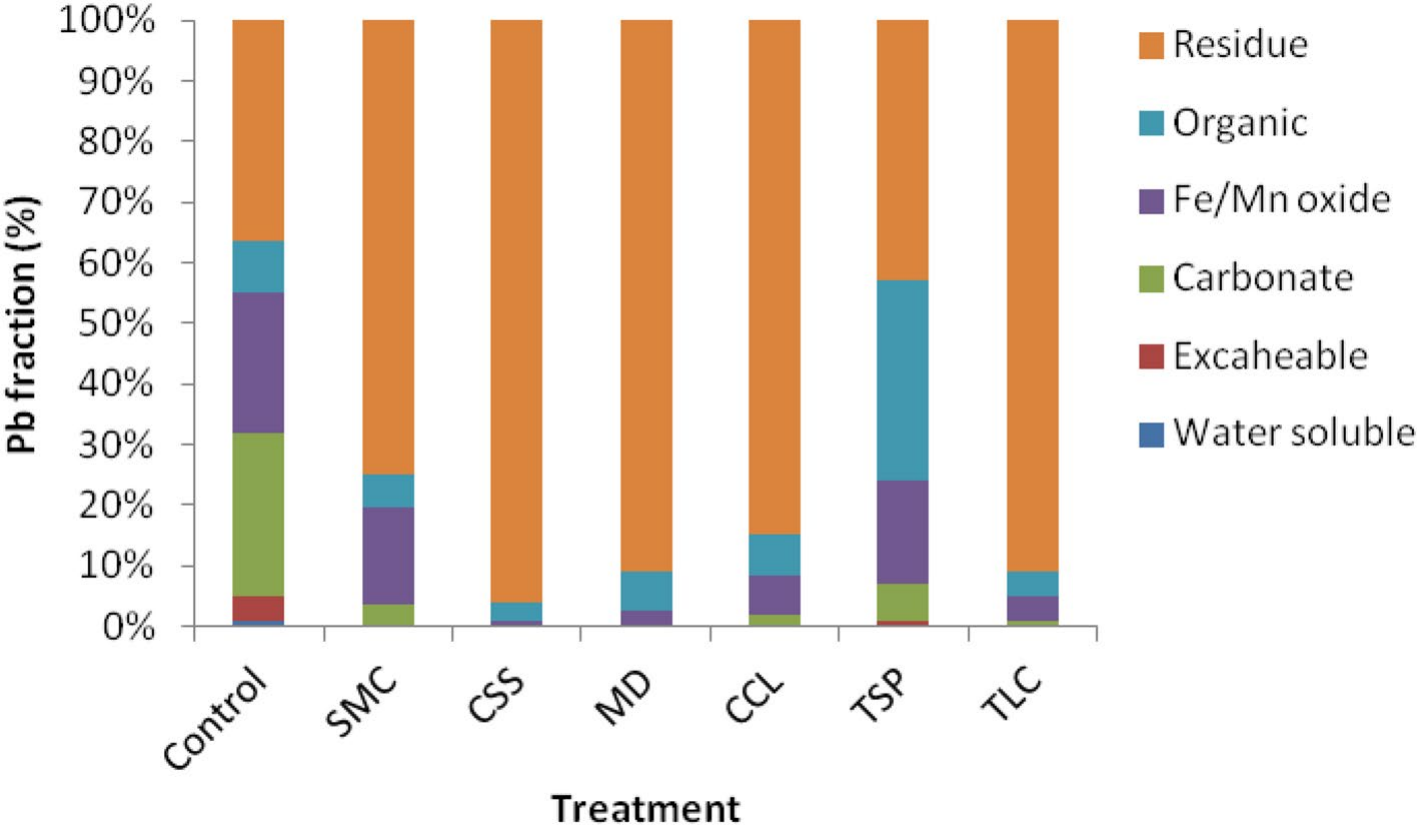
Average of Pb chemical fraction in the amendment-treated and untreated (control) mining wastes during the 8–10 year sampling period post treatment.

Exploring the Pb fractions and verifying the Pb transformations responsible for Pb stabilization and risk reduction by the treatments were critical for a better understanding of the treatment efficacies. In this study, a sequential extraction procedure combined with solid-phase speciation by the electron microprobe and microspectroscopy was used to identify the alteration of the Pb fractions induced by the biosolid amendments. In all cases, the residual Pb fractions in treated wastes significantly increased as a result of the treatments (Fig. 6, *p* < 0.05). In comparison with the control plot, the percentages in the residual fraction increased in the order: CSS (59%) > TLC (54%) = MD (54%) > CCL (48%) > SMC (38%) > TSP (6%). The residual Pb fraction, the least soluble or bioavailable fraction, could include pyromorphite or pyromorphite-like minerals. Increased residual Pb suggested the enhanced Pb transformation to relatively stable species by amendment treatments. Organic amendments had been reported to increase the insoluble or stable Pb fractions in rice paddy soil^23^ and calcareous soil^25^. Data also indicated that the formation of residual Pb was primarily from transformed labile Pb species including water-soluble, exchangeable, carbonate, and Fe/Mn oxide-associated fractions. As mentioned above, lead carbonate (PbCO_3_), a highly soluble and bioavailable Pb species, was identified as the primary Pb mineral in untreated wastes. This labile Pb mineral would facilitate Pb dissolution and was effectively transformed to the more stable species by the treatments. Xian^32^ and Zhang et al.^33^ studied metal fractions and the release from contaminated soils and indicated that water-soluble and exchangeable Pb were highly leachable or bioavailable. Exchangeable and carbonate Pb fractions were found to be responsible for Pb uptake by plants in contaminated alluvial soil and sewage sludge-treated soil^34,35^. Our finding that showed the Pb transformation from labile Pb species to more stable forms had validated the assessment of the health and ecological risk reductions in the treated wastes.

**Figure 7:**
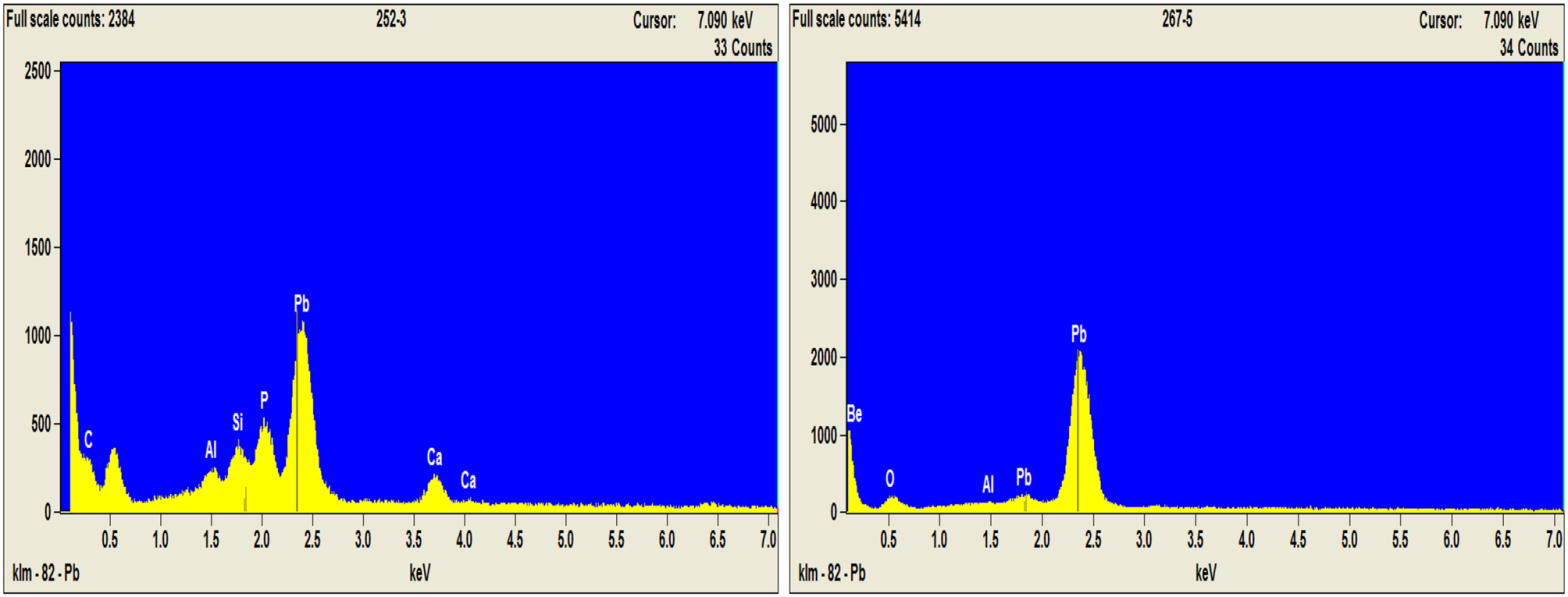
EDS patterns of Pb solids in selected amendment treated (SMC, left) and untreated (control, right) mining wastes during the 8–10 year sampling period post treatment.

Solid Pb particles in selected treated wastes were successfully identified by scanning electron beam microscopy (SEM) combined with energy-dispersive spectroscopy (EDS). The particles analyzed in treated wastes were found at a range of 20–40 µm size, containing the elements of Pb, P, Si, Ca or Al, while no P was present in untreated waste (Fig. 7). This solid speciation analysis verified the formation of solid Pb phosphates or pyromorphite-like minerals as induced by amendments and their association with clay minerals and carbonates present in the wastes. Nevertheless, less than 10% of the identified Pb solids in treated wastes were detected containing P or associated with Pb phosphates. This may suggest a low detection rate or partial transformation of pyromorphites or pyromorphite-like minerals in the presence of biosolids. Scheckel et al.^36^ indicated that pyromorphite formation was not facilitated or enhanced by organic amendment applications. The insignificant formation of pyromorphites in the high organic matter (OM) amended soil could be caused by (1) pyromorphite formation might be decreased by Pb-OM complexation; (2) pyromorphite crystal could be coated by organic coats that inhibited further pyromorphite formation and detection; or (3) organic coatings might help pyromorphite crystals move out of soil by enhanced colloidal transport^37^. In addition, the formation of pyromorphite could be inhibited in the presence of iron oxides and carbonates^38,39^. The surface coating of FePO_4_ or AlPO_4_ formed on the Pb solids may also prevent the formation and detection of pyromorphites^40^. Scheckel et al.^36^ also pointed out that it would be very difficult to distinguish the hexagonal crystals of pyromorphite from the arrays of hexagonal minerals. Pyromorphite-like minerals usually were heterogeneously distributed in the samples and in amorphous or poorly crystalline form without clear hexagonal crystal structure^12^. This solid-phase speciation analysis had verified the formation of pyromorphites or pyromorphite-like minerals after treatments and may also suggest an incomplete or partial Pb transformation to pyromorphites, which confirmed the results of chemical fractionation and partially accounted for the induced Pb immobilization from labile fractions to relatively stable species. Additional reactive mechanisms, such as chelation by organic function groups, surface adsorption and/or complexation by organic matter^12,24^, may also be involved in the Pb stabilizing processes.

## Conclusions

This field study showed that the application of six P-enriched biosolid amendments effectively restored the vegetation cover and reduced the bioacessibility, phytoavailability, and leachability of Pb in contaminated mining wastes. The treatments had no toxicological impact on soil microbial community. The efficacy of the treatments was maintained in the long-term and environmentally sound. Among the six amendments assessed, SMC and MD treatments appeared to be the most effective regarding the human health and ecological risk reductions. The chemical fractionation and solid speciation analysis verified the transformation of soil Pb from labile carbonate, exchangeable, and water-soluble fractions to relatively stable forms and confirmed the formation of pyromorphites or pyromorphite-like minerals induced by the treatments. The Pb transformations identified would be responsible or accounted for the health and ecological risk reductions by the amendments. This study demonstrated that in situ soil treatments using P-enriched biosolid amendments could be potentially applied as a long-term effective, economical, and ecologically sound remedial technology that stabilizes toxic Pb in mining waste sites and safeguards human health, water quality, and natural ecosystems from Pb contamination.
